# Factors affecting vaccine hesitancy among families with children 2 years old and younger in two urban communities in Manila, Philippines

**DOI:** 10.5365/wpsar.2019.10.2.006

**Published:** 2020-06-30

**Authors:** Julius Migriño, Billy Gayados, Karen Rachel Joyce Birol, Lorelie De Jesus, Christopher Willis Lopez, Winona Colleen Mercado, Jan-Mark Caezar Tolosa, Joeylyn Torreda, Glaze Tulagan

**Affiliations:** a College of Medicine, San Beda University, Manila, Philippines.; b School of Medicine and Public Health, Ateneo de Manila University, Pasig City, Philippines.

## Abstract

**Objective:**

The study aimed to determine the factors that influence vaccine hesitancy among parents and caregivers of children 2 years old and younger in selected urban communities in Manila, Philippines.

**Methodology:**

The study used a cross-sectional study design with a modified questionnaire adapted from the SAGE Working Group on Vaccine Hesitancy. Self-administered surveys were conducted in two highly urbanized barangays (smallest administrative divisions) in Manila, Philippines.

**Results:**

The survey was completed by 110 respondents, comprised mostly of 20–39-year-old mothers. Most respondents (95.5%) believed that vaccines are protective however vaccine hesitancy rates among the respondents reached 36.4%. Respondents who believed in the protective nature of vaccines were less likely to report vaccine hesitancy and were nine times less likely to refuse vaccination for their children because of negative media exposure. The main reasons identified for vaccine hesitancy were exposure to negative media information and concerns about vaccine safety. The main negative media information identified by the respondents was related to the dengue vaccine, Dengvaxia®. Health-care workers and political leaders were the main supporters of vaccination in the community.

**Discussion:**

The recent events surrounding the Dengvaxia® controversy contributed to a decrease in vaccine confidence. The role of mass media in vaccine hesitancy was highlighted in this study, supporting previous evidence that vaccine-hesitant parents tend to be more susceptible to media reports. The lack of association between sociodemographic factors and vaccine hesitancy implies that the determinants of vaccine hesitancy can be highly varied depending on context and setting.

Immunization has been one of the most important strategies in public health, and it is one of the most cost-effective interventions that lead to improvement of global health outcomes. Childhood mortality from measles and tetanus has drastically decreased through effective national immunization programmes, (*1*) and it is estimated that 2–3 million deaths per year are prevented through vaccination. (*2*) However, for immunization strategies to make significant strides in curbing morbidity and mortality, uptake rates for vaccines need to reach critical levels. Measles vaccination, for example, needs to reach a population rate of around 83–94% to elicit herd protection and prevent outbreaks. (*3*, *4*) While global trends show an increase in the vaccination rates for specific antigens, there have been resurgences or increases in the rates of some vaccine-preventable diseases (e.g. measles, circulating vaccine-derived poliovirus) in the past few years. (*5*) Beginning in early 2019 in the Philippines, the Department of Health (DOH) declared measles outbreaks in at least six regions – Davao Region, Metro Manila, Central Luzon, Calabarzon, Western Visayas and Central Visayas. (*6*) There was a staggering eightfold increase in the incidence rate from late 2017 to 2018, and the trend continued with more cases of measles reported in the first quarter of 2019 compared to all of 2018. (*7*)

In November 2017, a media frenzy erupted. One year after the Philippines initiated a mass vaccination campaign with the first licensed dengue vaccine (Dengvaxia®) that reached around 800 000 schoolchildren, Sanofi Pasteur, the manufacturer of Dengvaxia®, revealed that the vaccine potentially increased the risk of severe dengue in children who had never been infected with dengue before vaccination. (*8*) The DOH and several studies identified the controversy that arose as one of the probable reasons for the loss of vaccine confidence in the Philippines, (*8*-*11*) which could have contributed to the rise in measles cases in 2018. (*8*-*10*)

Vaccine hesitancy is defined as a “delay in acceptance or refusal of vaccines despite availability of vaccination services.” (*12*) While the reasons for delays or refusals to accept vaccines are complex, the Strategic Advisory Group of Experts (SAGE) technical working group has accepted two working models regarding the determinants of vaccine hesitancy. (*12*) The 3Cs model, composed of complacency, convenience and confidence, is a simpler intuitive model. The Working Group Matrix (“Matrix”) is more comprehensive and aims to categorize the determinants of vaccine hesitancy into three major groups: contextual influences (influences arising due to historic, sociocultural, environmental, health system/institutional, economic or political factors); individual and group influences (influences arising from personal perception of the vaccine or influences of the social/peer environment); and vaccine/vaccination-specific issues (issues directly related to vaccines or vaccination). (*12*, *13*) It is clear that vaccine hesitancy is a problem, and addressing its determinants using either model is key at the policy level to prevent vaccine hesitancy and the emergence of outbreaks of vaccine-preventable diseases in groups with low vaccination rates. (*14*)

Evidence about factors associated with vaccine hesitancy in the Philippines is lacking. The subject is timely due to the recent Dengvaxia® controversy, a subsequent decrease in vaccine confidence and the more recent outbreak of measles in the country. The objective of this study was to determine the factors associated with vaccine hesitancy in urban communities in Manila, Philippines. Identifying and understanding these factors are crucial to inform interventions that can address the issues and lead to increased vaccination rates.

## Methods

**Figure 1:**
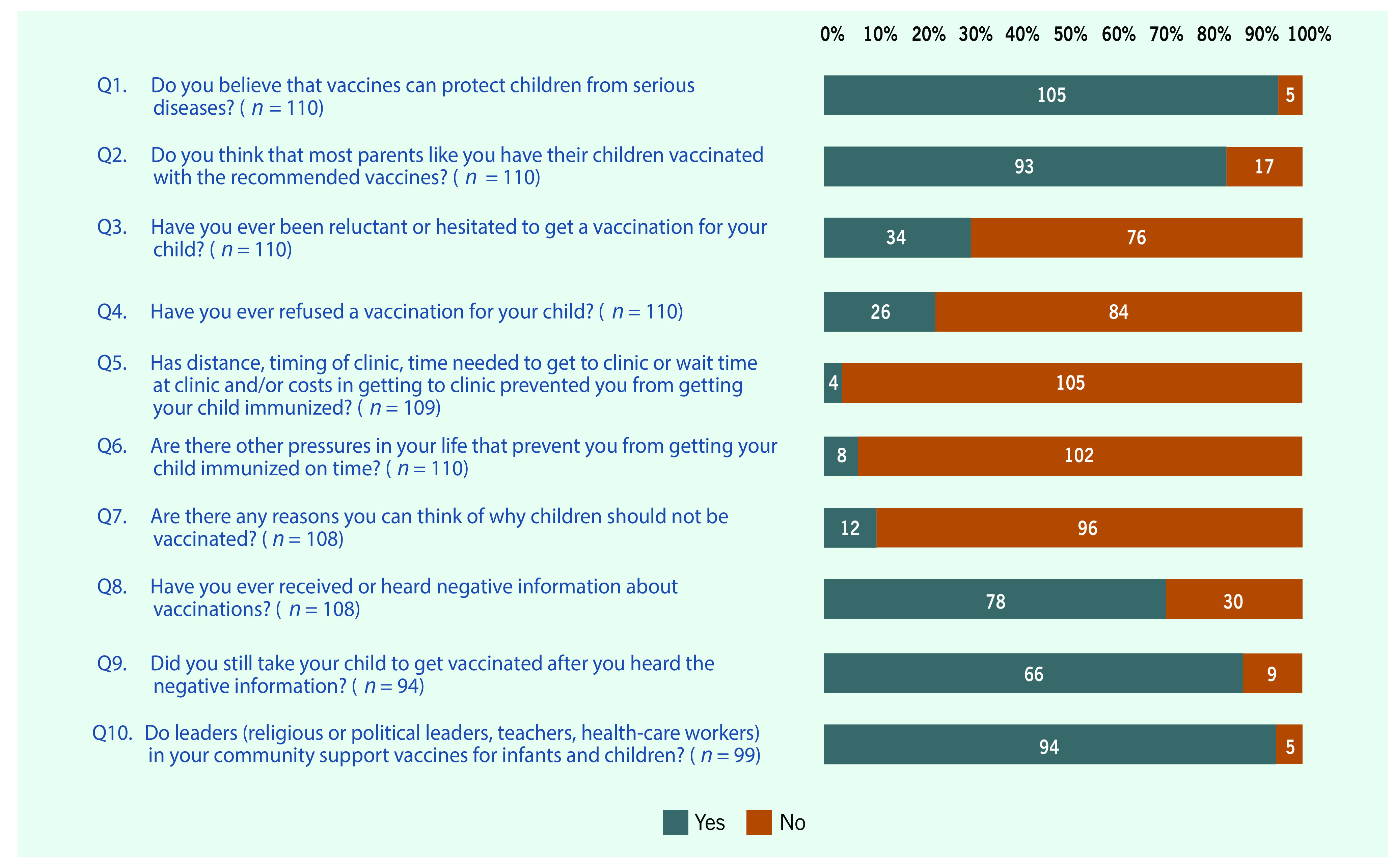
**Summary of survey responses of parents or caregivers of children 2 years old or younger in two barangays in Manila, Philippines (n = 100)**

We developed a survey that was adapted from a previous vaccine hesitancy survey. (*15*) The revised questionnaire consisted of 10 core closed questions to assess vaccine hesitancy of parents and caregivers at a community level (**Fig. 1**). Probe questions were also included for questions 4, 7, 8 and 10 to determine specific reasons respondents answered “yes” to these questions (**Table 1**). The questionnaire was translated into Filipino and was back-translated into English for the purposes of content validation and pretesting before administration. Data were collected using self-administered questionnaires.

[**insert** Figure 1]

**Table 1:** Answers to probe questions from respondents who answered “Yes” to core questions Q4, Q7, Q8 and Q10

Q4. Have you ever refused a vaccination for your child? (*n* = 26)		
Heard or read negative media	18	69.2%
Did not think the vaccine was safe or concerned about the side-effects	12	46.2%
Did not think vaccine was effective	5	19.2%
Someone else told me that vaccine was not safe	5	19.2%
Did not think it was needed	4	15.4%
Someone else told me they/their child had a bad reaction	3	11.5%
Did not know where to get good/reliable information	1	3.8%
Had a bad experience or reaction with previous vaccination	1	3.8%
Others	1	3.8%
-		
Q7. Are there any reasons you can think of why children should not be vaccinated? (*n* = 12)		
They choose not to vaccinate	5	41.7%
They do not feel welcome at the health service	1	8.3%
Health services do not reach them	1	8.3%
-		
Q8. Have you ever received or heard negative information about vaccinations? (*n* = 78)		
“Dengvaxia®”	59	75.6%
“Vaccines are deadly”	1	1.3%
-		
Q10. Do leaders (religious or political leaders, teachers, health-care workers) in your community support vaccines for infants and children? (*n* = 94)		
Health-care worker	76	76.8%
Political	68	68.7%
Teacher	23	23.2%
Religious	18	18.2%

*Note: respondents may give more than one answer

The study sites were two small and highly urbanized barangays (smallest administrative divisions) situated in the district of San Miguel in Manila, Philippines. These sites were purposively selected based on ongoing health services collaboration between San Beda University College of Medicine and the barangays. A sample size of 109 was calculated using OpenEpi (*16*) based on the estimated number of families with children 2 years old or younger from the study sites (*n* = 154, sample proportion = 0.32, confidence level = 95%, α = 0.05). (*17*) Purposive recruitment of eligible respondents was done with the help of barangay health workers, as well as snowball sampling from previous respondents, until the minimum sample size was accomplished. Sampling was started at the house nearest the health centre and then at the nearest house with an identified eligible respondent. Parents and caregivers aged 18 years or older of at least one child 2 years old or younger who had lived in the study sites for at least one year were eligible to be included in the study. Written informed consent containing the study’s brief introduction, nature of risks and benefits, provision for confidentiality and voluntary nature was collected from each participant before the survey. Parents and caregivers of children who had contraindications to routine vaccinations (e.g. severe allergic reactions to previous exposure, immunocompromised status) were excluded from the study. Ethical approval of the study was provided by San Beda University Office for Research and Innovation.

All data were entered in Microsoft Excel and then coded and analysed using StataCorp. 2013. (Stata Statistical Software: Release 13. College Station, TX) Categorical variables were summarized using frequencies and percentages; χ^2^ analyses with Phi coefficient post-hoc tests were used to determine correlations with and among the factors associated with vaccine hesitancy and refusal. Binary logistic regression was used to determine the odds ratio (OR) and 95% confidence intervals (CI).

### Ethics Statement

The study was reviewed and approved on 18 January 2019 by the San Beda University Office for Research and Innovation. Permission and approval were obtained from the Division of Planning and Coordination, Manila Health Department, City of Manila, approval number 8159759.

## Results

A total of 150 houses were identified with eligible respondents; 31 of them were excluded from the sampling frame (either nobody was home or the children were older than 2 years). A total of 119 respondents completed the survey (100% response rate); however, only 110 responses were included in the final data due to incomplete survey or informed consent information. **Table 2** summarizes the demographic characteristics of the respondents. Most of the respondents were women (81.8%), and most were the mothers (73.6%). Fathers accounted for 17.3% of the total respondents. The median age of the respondents was 29 years old (interquartile range = 25–33). Almost 65% of the respondents finished at least some high school education, and 34.6% had some college-level education. The predominant religion was Roman Catholic (76.4%). The reported monthly household incomes varied, but 87.3% reported that their monthly household income was less than 20 000 Philippine pesos.

**Table 2:** Answers to probe questions from respondents who answered “Yes” to core questions Q4, Q7, Q8, Q10

-	Frequency	Percentage
**Gender**		
Female	90	81.8%
Male	20	18.2%
**Relationship to child**		
Mother	81	73.6%
Father	19	17.3%
Grandmother	8	7.3%
Grandfather	1	0.9%
Aunt	1	0.9%
**Age range (years)**		
less than 20	10	9.1%
20–29	50	45.5%
30–39	36	32.7%
40–49	6	5.5%
50–59	3	2.7%
60 and above	5	4.6%
**Educational attainment**		
Elementary school	11	10.0%
High school	60	54.6%
College	38	34.6%
Vocational school	1	0.9%
**Religion**		
Roman Catholic	84	76.4%
Iglesia ni Cristo	3	2.7%
Christian, other denomination or nondenominational	11	10.0%
Muslim	11	10.0%
Others	1	0.9%
**Household monthly income (Philippine pesos)**		
less than 5 000	24	21.8%
5000 to < 10 000	33	30.0%
10 000 to < 15 000	24	21.8%
15 000 to < 20 000	15	13.6%
20 000 and above	14	12.7%

1 USD = 50.4 PHP, at the time of publication

**Fig. 1** summarizes the answers of the respondents to the survey questionnaire. Almost all (95.5%) respondents believed that vaccines are protective to children, and many (84.6%) believed that most parents have their children vaccinated with recommended vaccines. Ninety-six per cent of respondents reported that financial and logistical concerns have not prevented them from getting their children vaccinated; 92.7% mentioned that other pressures in life have not prevented them from getting their children vaccinated on time. Almost 11% of respondents believed that there could be reasons why children should not be vaccinated; 41.7% of them believed that they can choose not to vaccinate. The majority (72.2%) of respondents had heard negative information about vaccinations, and of these, 75.6% reported hearing negative information about Dengvaxia®. Despite this, 88.0% of respondents who reported receiving negative information about vaccinations said that they would still take their children to get vaccinated. A large majority (95.0%) agreed that community leaders support child vaccination. Health-care workers and political leaders were identified as top vaccination advocates (76.8% and 68.7%, respectively) followed by teachers and religious leaders (23.2% and 18.2%, respectively).

Thirty-one per cent reported hesitating to give at least one vaccination to their children, and 23.7% outright refused at least one vaccination for their children. Cumulatively, 36.4% of the respondents either hesitated or refused to give at least one vaccination (or both) to their children. Respondents who hesitated to have their children receive at least one vaccination were also 16.7 times more likely to have refused least one vaccination for their children (OR = 16.7, 95% CI = 5.7–49.0, *P* < 0.001). A χ^2^ analysis with Phi coefficient post-hoc test revealed that respondents who have hesitated to have their child vaccinated were (1) less likely to believe that vaccines protect children from serious diseases (χ^2^(1) = 9.2, *P* < 0.01, Φ = −0.3), and (2) more likely to have experienced significant life events that prevented them from having their children vaccinated on time (χ^2^(1) = 9.7, *P* < 0.01, Φ = 0.3). There were no significant associations between vaccine hesitancy and demographic data (respondent’s age, gender, educational attainment, religion, income category and relationship of the respondent to the child).

The main reasons for refusing to have their child vaccinated are shown in **Table 1**. The primary reason for vaccine refusal was negative information from the media (69.2%), followed by concerns about the safety of vaccines (46.2%). There was a strong association between these reasons (χ^2^(1) = 68.8, *P* < 0.001, Φ = 0.8). Further analysis revealed that respondents who believed in the protective nature of vaccines were 9.0 times less likely to refuse vaccination for their children because of negative media exposure (OR = 0.11, 95% CI = 0.017–0.72, *P* < 0.05, pseudo R^2^ = 0.12) and 6.3 times less likely to refuse vaccination for their children because of vaccine safety concerns (OR = 0.16, 95% CI = 0.024–1.1, *P* < 0.1, pseudo R^2^ = 0.07).

Other reasons for refusing to have their children vaccinated at least once included the beliefs that vaccines were not effective (19.2%) and that vaccines were not safe (19.2%), doubts about the need for vaccination (15.4%), someone telling them about adverse reactions following vaccinations (11.5%), having a bad experience during previous vaccinations (3.8%) and not knowing where to get reliable information (3.8%). There were no significant associations between reasons for vaccine refusal and respondent’s age, gender, educational attainment, religion, income bracket and relationship to child.

## Discussion

This study identified the presence of vaccine hesitancy in about one third of the respondents from two highly urbanized communities in Manila, Philippines. The main reasons for refusing at least one vaccination for their children were negative media information and concerns about the safety of vaccines and their side-effects; the main negative media information identified by the respondents was related to the Dengavaxia® vaccine.

Vaccine hesitation is a threat to individuals and also to public health. In the Philippines, it has been suggested that the recent events surrounding the dengue vaccine Dengvaxia® has contributed to a decrease in vaccine confidence; (*9*, *18*) however, data supporting this contention are lacking particularly in many low- and middle-income countries. Many reasons have been identified as potential sources of vaccine hesitancy, and beliefs and attitudes towards vaccine efficacy and safety are among them. (*14*, *15*) One study reported that vaccine hesitancy was found to be low in parents who perceive vaccination as important. (*19*) This is consistent with the results of our study that showed respondents who believe in the protective nature of vaccines were less likely to have hesitated or refused vaccination for their child. Circumstantial life events surrounding vaccination have also been identified in literature as potential factors of vaccine hesitancy, where parents attach significance to events such as their child’s birth timing, sleep patterns or behaviour, rather than rely on a science-based approach to health care, including immunization. (*14*) This was consistent with our study findings: respondents with some form of significant event during vaccination periods were more likely to be vaccine-hesitant.

Mass media, such as newspapers, television, radio, the Internet and social media, has contributed to the growing problem of vaccine distrust primarily by over-reporting adverse events of immunization. (*20*-*22*) A compounding factor is that vaccine-hesitant parents tend to be more susceptible to media reports, whether verified or not, (*21*, *23*) and they frequently rely on the Internet as their source for vaccination information. (*14*) This phenomenon has been characterized in this study: there was a significant positive association between exposure to negative media information about vaccines and vaccine hesitancy among the study population. Negative media information was positively correlated with safety concerns that correlated with refusal to have children vaccinated at least once in the past. The Dengvaxia® issue in the Philippines was propagated in all types of media beginning in late 2017, and three quarters of study respondents who reported having heard negative information about vaccines said they had heard negative information about Dengvaxia®. Most of the media information was reported on Internet news sites, newspapers and social media that contained reports of adverse events during or after the vaccination campaign, including official statements on fatalities and growing distrust of the vaccine. (*10*, *24*-*26*) These events are not unique to the Philippines. In 2013, Viet Nam experienced a similar story surrounding Quinvaxem® (diphtheria, tetanus, whooping cough, hepatitis B, *Haemophilus influenzae* type B pentavalent vaccine), where some young infants allegedly had allergies, seizures or reduced muscle tone shortly after receiving the vaccine. Vaccine hesitancy and refusal, and the resulting decrease in vaccine coverage, were linked to extensive print and online media campaigns of the adverse effects of immunization. (*20*) The controversy led to loss of public trust, and parents had to wait for another pentavalent vaccine to become available. (*20*)

The results of our study suggest vaccine hesitancy is an issue for parents and caregivers of children 2 years old and younger regardless of age, gender, educational attainment, religion, income bracket or relationship to the children. Some international effects of gender inequality (*27*) on vaccination attitudes and practices (including vaccine hesitancy), such as men’s purported distrust towards vaccinations and women’s greater motivation to access health services for their children, did not seem to be present in the study population. One study suggested that educational levels and religious affiliations of caregivers may influence vaccine hesitancy; however, we did not find this in our study. The SAGE Working Group study (*12*) noted that the level of education may both promote and impede vaccine acceptance depending on the setting. Because the determinants of vaccine hesitancy can be highly varied, contextualization of determinants in each setting (and not general assumptions) is advised by the experts before interventions can be devised. (*12*)

The purposive nature of the study site and convenience sampling method in respondent selection limits the generalizability of the study to similar study sites (i.e. small, highly urbanized communities); however, literature suggests that different communities have different determinants of vaccine hesitancy. There is always the need to identify these determinants that collectively influence vaccination beliefs and practices and not solely rely on generalizations. (*12*) Another limitation of the study is the exclusion of parents and caregivers of children who have any contraindications to routine vaccination; contraindication to one vaccine does not necessarily mean contraindication to all vaccines, and this important population subgroup might have been missed in the study. Potential biases that may have affected the results include recall bias and social desirability bias.

The results of this study suggest that vaccine hesitancy might be addressed by a multistakeholder approach in the community. The role of political and religious community leaders in supporting vaccination strategies appears to be evident. The role of health workers needs to be re-emphasized and strengthened; they were the most commonly cited advocates for vaccination in this study. In a previous study, they were found to be the most influential persons addressing vaccine hesitancy. Empowering and mobilizing health workers to take an active role in promoting accurate and timely information on the benefits of immunization and allaying the community’s fears and distrust of vaccines is still the most important strategy. (*21*)
